# Sports injuries among adolescent basketball players according to position on the court

**DOI:** 10.1186/1755-7682-6-5

**Published:** 2013-02-13

**Authors:** Franciele Marques Vanderlei, Fabio Nascimento Bastos, Ítalo Ribeiro de Lemes, Luiz Carlos Marques Vanderlei, Jayme Netto Júnior, Carlos Marcelo Pastre

**Affiliations:** 1 Departamento de Cardiologia, UNIFESP – Univ Federal de São Paulo, Rua Roberto Simonsen, 305, São Paulo, Brazil; 2 Departamento de Fisioterapia, UENP – Univ Estadual do Norte do Paraná, Jacarezinho, PR, Brazil; 3 Faculdade de Ciências e Tecnologia, Laboratório de Fisioterapia Desportiva – LAFIDE, UNESP – Univ Estadual Paulista, Presidente Prudente, SP, Brazil; 4 Faculdade de Ciências e Tecnologia, Programa de Pós-Graduação em Fisioterapia, Laboratório de Fisioterapia Desportiva – LAFIDE, UNESP – Univ Estadual Paulista, Presidente Prudente, SP, Brazil

**Keywords:** Trauma in athletes, Risk factors, Morbidity surveys

## Abstract

**Background:**

The participation of children and adolescents in sports, including basketball, is becoming increasingly common, and this increased involvement raises concerns about the potential risk of sports injuries.

**Objective:**

To analyze the occurrence of sports injuries among young basketball players according to their position on the court and to associate these injuries with risk factors.

**Method:**

A retrospective, epidemiological study. A sample consisting of 204 basketball players with a mean age of 14.33 ± 1.19 years participated in the study. The players were interviewed using a reported condition questionnaire containing anthropometric and training data as well as information on injuries during the previous 12 months.

**Results:**

The frequency of injury was highest among the shooting guards (47.8%), followed by the centers (34.8%) and point guards (17.4%). Among the 204 participants, 40 players reported a total of 46 injuries, representing 0.22 injuries per participant and 1.15 injuries per injured participant. For the shooting guards and centers, statistically significant differences between injured and non-injured players were found related to age, weight, height, length of time in training and number of weekly practice hours (p < 0.05). For point guards, a statistically significant difference between injured and non-injured players was found based on weight alone (p < 0.05).

**Conclusion:**

The occurrence of injuries among basketball players was low. Injuries were associated with both intrinsic and extrinsic factors among shooting guards and centers, whereas injuries were only associated with weight among point guards.

## Background

Basketball is a popular sport played worldwide [1] which requires dynamic, explosive actions. The constant practice of this sport involves repetitive motor actions and excessive joint load, which increases the vulnerability to injury [2]. Although practiced by millions of individuals, including adolescents [1,2], there is a lack of information to enable the determination of associations between sports injuries and personal and training characteristics as well as position on the court.

Studies have shown that the most common injuries in basketball affect the lower limbs, specifically the knee and ankle sprains [3,4]. This is due to the fact that basketball is a sport characterized by actions involving running, changes of direction, lateral movements, jumping, and, in particular, the impact of landing, which are conducive to the onset of injuries in these regions [4].

The aim of this study was to analyze the occurrence of injuries among adolescent basketball players according to position on the court and to associate these injuries with intrinsic (age, height and weight) and extrinsic factors (length of time in training, and number of hours practice per week).

## Materials and Methods

This study was approved by the local Human Research Ethics Committee (protocol: 08/2010). Two hundred four randomly selected basketball players (62 females and 142 males) in the city of Presidente Prudente (State of São Paulo, Brazil) with a mean age of 14.33 ± 1.19 years participated in the study. All the volunteers who were part of the sample attended Sports Initiation Schools provided by the Municipal Sports Secretariat in the city, and presented a low level of training and competitiveness. Data were collected retrospectively using a modified reported condition questionnaire [5], which included personal data (gender, age, weight and height), training data (length of time in training, and number of hours practice per week) and information on the occurrence of injury.

We included volunteers from local authority sports schools provided by the Municipality of Presidente Prudente, aged up to 18 years, who had practiced basketball for at least one year and who accepted the invitation to participate in the study and whose consent was authorized by a legal guardian. It was stipulated that, once selected, volunteers who experienced difficulty understanding the questions of the survey or who presented injuries at other times unrelated to training and/or sports competitions would be excluded, since these factors could interfere with the quality of response or the reliability of the findings. In the event, none of those previously selected was excluded.

A sports injury was considered to be any physical complaint resulting from training and/or competition which limited the participation of the individual for at least one day, regardless of the need for medical attention [6,7]. For the purposes of analysis, the participants were grouped separately, based on their respective positions on the court: shooting guard, center and point guard.

The data were analyzed using descriptive statistics. Depending on the normality of the data, either the *Student’s t-test* for unpaired data or the *Mann–Whitney test* was used. *Odd ratios* (OR) and 95% confidences intervals were calculated for the comparison between genders. The level of significance was set at 5% (p < 0.05) for all analyses.

## Results

No significant differences were found between genders with regard to the position on the court (shooting guard – OR: 1.07; 95% CI: 0.36 to 3.17; center – OR: 2.69; 95% CI: 0.83 to 8.75; point guard – OR: 6.00; 95% CI: 0.95 to 38.08). The greatest frequency of injury was found among the shooting guards, followed by the centers and point guards (Table 1).

**Table 1:** Distribution of injured athletes, reported injuries, rate of injury per athlete and rate of injury per injured athlete

**Variables**		**Position**		**Total (n = 204)**
	**Shooting guard (n = 103)**	**Center (n = 72)**	**Point guard (n = 29)**	
**Injured athletes**	18 (45%)	15 (37.5%)	7 (17.5%)	40 (100%)
**Reported injury**	22 (47.8%)	16 (34.8%)	8 (17.4%)	46 (100%)
**Rate of injury per athlete**	0.21	0.22	0.27	0.22
**Rate of injury per injured athlete**	1.22	1.06	1.14	1.15

**Note:** Rate of injury per athlete = total number of injuries divided by total number of athletes interviewed; rate of injury per injured athlete = total number of injuries divided by total number of injured athletes.

Statistically higher values were found relating to age, height, length of time in training and number of weekly practice hours among the shooting guards and centers who had suffered injuries in the previous 12 months in comparison to non-injured participants. Regarding weight, heavier athletes in all positions reported more injuries in comparison to lighter athletes (Table 2).

**Table 2:** Values of mean, standard deviation and median of anthropometric data and training variables according to position and occurrence of injury

**Variables**	**Position**	**Injury**		**p-value**
		**Present (n = 164)**	**Absent (n = 46)**	
**Age (years)**	Shooting guard	15.54 ± 1.18 (15.00) *	13.95 ± 0.98 (14.00)	0.0001
	Center	15.37 ± 0.71 (15.00) *	14.15 ± 1.20 (14.00)	0.0003
	Point guard	14.33 ± 0.70 (14.00)	14.30 ± 1.18 (14.00)	0.89
**Weight (Kg)**	Shooting guard	67.44 ± 14.29 † (68.80)	58.34 ± 10.11 (58.00)	0.009
	Center	74.91 ± 15.34 † (72.00)	62.62 ± 14.82 (58.00)	0.004
	Point guard	67.24 ± 13.32 † (62.00)	58.33 ± 8.75 (56.00)	0.03
**Height (m)**	Shooting guard	1.73 ± 0.10 † (1.71)	1.65 ± 0.07 (1.66)	0.003
	Center	1.77 ± 0.07 † (1.77)	1.67 ± 0.07 (1.69)	0.0001
	Point guard	1.72 ± 0.09 (1.71)	1.66 ± 0.05 (1.68)	0.10
**Training duration (years)**	Shooting guard	3.84 ± 1.93 (3.00) *	1.91 ± 1.30 (1.50)	0.0001
	Center	3.87 ± 1.54 (3.00) *	2.57 ± 1.64 (2.00)	0.003
	Point guard	2.88 ± 1.53 (3.00)	2.30 ± 1.22 (2.00)	0.30
**Weekly hours of practice**	Shooting guard	5.86 ± 2.46 (6.00) *	4.89 ± 2.36 (4.50)	0.02
	Center	6.12 ± 2.17 (6.00) *	4.49 ± 2.47 (4.00)	0.005
	Point guard	6.72 ± 5.35 (4.50)	4.91 ± 1.49 (6.00)	0.89

* Significant difference in relation to non-injured participants (*Mann–Whitney test*); † Significant difference in relation to non-injured participants (*Student’s t-test for unpaired*).

## Discussion

The main findings demonstrate a greater frequency of injuries among shooting guards, followed by centers and point guards. Moreover, individual and training characteristics may be associated with risk factors for shooting guards and centers, and body mass may be a risk factor for all positions.

In adolescent basketball players the highest frequency of injuries was among shooting guards, followed by centers and point guards. Cohen et al., 1999 [8] found no significant differences regarding the occurrence of injuries among the different positions of adult players. Moreira et al., 2003 [9] analyzed professional adult players with a mean age of 24.5 years and found a greater frequency of injuries among centers, followed by shooting guards and point guards. The differences between research findings may be related to the age group studied.

While there are five positions relating to both offense and defense, the point guard is responsible for the transition from defense to offence, with speed and agility and creating opportunities for his/her teammates to prepare the shots. This style of play predisposes the point guard to sprained ankles [6,9]. Shooting guards are responsible for the preparation of the shot and therefore play more in terms of volume, blending agility, speed, strength and power [9,10]. As decisive offensive players, shooting guards suffer more checking, which may explain the occurrence of injuries. Centers are responsible for shots within the key, which involve disputes for defensive and offensive rebounds and shots that require the use of brute force when vying for space. As a position in which constant physical contact occurs, the majority of injuries are more traumatic [6,7,9].

The biotype of players is considered to be a determining factor regarding the position played on the court [11]. The study demonstrated that individual characteristics of the players may be related to the occurrence of injuries. For point guards, height was not associated with injuries. The main function of these players is to set up the offensive actions and advance the ball with speed and agility. While the occurrence of injury was not associated with height, shorter players are required to be faster in order to achieve these tasks [11,12]. For centers and shooting guards, taller athletes reported more injuries than shorter athletes. Athletes with offensive functions play closer to the basket. Thus, a greater height is needed in order to perfect the handling of rebounds and score a field goal. These actions require greater physical contact, which predisposes players to injury and explains the greater occurrence of injuries among taller players [11].

Regarding body mass, a greater frequency of injuries occurred among heavier athletes. Point guards normally play further away from the net and consequently execute fewer jumps and absorb less impact [11]. Thus, the dynamics of this position does not explain the greater occurrence of injury among heavier point guards; however, body weight is no less an important factor with regard to the occurrence of injuries among these players. Centers, on the other hand, need greater weight for blocking and occupying space close to the basket during offensive and defensive rebounds. The absorption of impact during the constant jumping and landing promotes the occurrence of injuries among heavier players. Thus, body mass should be taken into consideration within the dynamics of the game in all positions that require force, speed and muscle strength [11].

The results show that the average age of the players is associated with injury risk factors. The reasons for this occurrence are related to greater inclusion in sports due to specific interests, which occurs with the development age, associated with the characteristics of body composition, muscular strength and explosiveness and enthusiasm to compete in the chosen sport [13]. It is believed that the growth occurring as the players’ age increases is closely related to increased risk of injury due to the emergence of a combination of multiple factors, such as, increased musculotendinous strain, decreased epiphyseal strength and decline in bone mineralization [14,15].

In relation to length of time in training, we observed that players who train longer have more injuries compared with those who train less. The increased the exposure may be related to an increased risk of injury due to repetitive and cumulative trauma [16].

The sample size according to position was small and the analyses of the study were only able to infer characteristics that may influence the occurrence of injury. Another limitation of the study was the retrospective factor. However, the results of the study underline the need for programs geared to strategies that can control risk factors and reduce the occurrence of injuries, taking the player’s position into consideration.

## Conclusion

The findings demonstrate a greater frequency of injuries among shooting guards, followed by centers and point guards. Moreover, individual (age, height and weight) and training (length of time in training and number of practice hours per week) characteristics may be associated with intrinsic and extrinsic risk factors for shooting guards and centers, and body mass may be a risk factor for all positions.

## Competing interests

The authors declare that they have no competing interests.

## Authors’ contributions

CMP and FMV conceived of the study, participated in its design and coordination and helped to draft the manuscript. CMP, FMV, LCMV, FNB, IRL and JNJ performed the statistical analysis and interpretation of data and prepared the draft manuscript. All authors participated in the design of the study and in critical review of the manuscript. All authors read and approved the final manuscript.

## References

[B1] Borowshi LA Yard EE Fields SK The epidemiology of US High School Basketball Injuries, 2005–2007 Am J Sports Med 2008 36 12 2328 2335 10.1177/0363546508322893 18765675

[B2] Gaca AN Basketball injuries in children Pediatr Radiol 2009 39 12 1275 1285 10.1007/s00247-009-1360-0 19774373

[B3] Adirim TA Barouh A Common orthopaedic injuries in young athletes Current Paediatrics 2006 16 3 205 210 10.1016/j.cupe.2006.03.001

[B4] Dick R Hertel J Agel J Grossman J Marshall SW Descriptive epidemiology of collegiate men’s basketball injuries: National collegiate athletic association injury surveillance system, 1988–1909 through 2003–2004 J Athl Train 2007 42 2 194 201 17710167 PMC1941286

[B5] Pastre CM Carvalho Filho G Monteiro HL CR Lesões desportivas no atletismo: comparação entre informações obtidas em prontuários e inquéritos de morbidade referida Rev Bras Med Esporte 2004 10 1 1 8

[B6] Gantus MC Assumpção JD Epidemiology of the injuries of the locomotor system in basketball athletes Acta Fisiátrica 2002 9 2 77 84

[B7] Meeuwisse WH Sellmer R Hagel BE Rates and risks of injury during Intercollegiate Basketball Am J Sports Med 2003 31 3 379 385 12750130 10.1177/03635465030310030901

[B8] Cohen M Abdalla RJ Ejnisman B Lesões musculo-esqueléticas no basquete masculino Aparelho Locomotor 1999 3 18 21

[B9] Moreira P Gentil D Oliveira C Prevalência de lesões na temporada 2002 da Seleção Brasileira Masculina de Basquete Rev Bras Med Esporte 2003 9 5 258 262

[B10] Abdelkrim NB Fazaa SE Ati JE Time-motion analysis and physiological data of elite under-19-year-old basketball players during competition Br J Sports Med 2007 41 2 69 75 10.1136/bjsm.2006.032318 17138630 PMC2658931

[B11] Neto Paiva A César MC Body composition assessment in male basketball players in Brazilian National Basketball League 2003 Rev Bras Cine Des Hum 2005 7 1 35 44

[B12] Moreira P Prevalence of injuries of Brazilian Basketball Nation Team (principal and sub-21) during 2003 season R Bras Ci e Mov 2006 14 2 65 72

[B13] Deitch JR Starkey C Walters SL Injury risk in professional basketball players: a comparison of Women’s National Basketball Association and National Basketball Association athletes Am J Sports Med 2006 34 1077 1083 10.1177/0363546505285383 16493173

[B14] Baxter-Jones ADG Thompson AM Malina RM Mafulli MD Growth and maturation in elite young athletes Sports Med Arthrosc Review 2002 10 1 42 49 10.1097/00132585-200210010-00007

[B15] Caine D Maffulli N Caine C Epidemiology of injury in child and adolescent sports: injury rates, risk factors, and prevention Clin Sports Med 2008 27 1 19 50 10.1016/j.csm.2007.10.008 18206567

[B16] Agel J Olson DE CAQ Primary Care Sports Medicine Descriptive epidemiology of collegiate women’s basketball injuries: national collegiate athletic association injury surveillance system, 1988–1989 through 2003–2004 J Athl Train 2007 42 202 210 17710168 PMC1941290

